# Left atrial function index (LAFI) and outcome in patients undergoing transcatheter aortic valve replacement

**DOI:** 10.1007/s00392-022-02010-5

**Published:** 2022-03-23

**Authors:** Jasmin Shamekhi, Thi Quynh Anh Nguyen, Helen Sigel, Oliver Maier, Kerstin Piayda, Tobias Zeus, Baravan Al-Kassou, Marcel Weber, Sebastian Zimmer, Atsushi Sugiura, Nihal Wilde, Malte Kelm, Georg Nickenig, Verena Veulemans, Alexander Sedaghat

**Affiliations:** 1 Heart Center Bonn, Department of Medicine II, University Hospital Bonn, Venusberg-Campus 1, 53127 Bonn, Germany; 2 Heart Center, Department of Cardiology, University Hospital Düsseldorf, Düsseldorf, Germany

**Keywords:** Left atrial function index, TAVR, Transcatheter aortic valve replacement, LAFI

## Abstract

**Background:**

Clinical data regarding the association between the left atrial function index (LAFI) and outcome in patients undergoing transcatheter aortic valve replacement (TAVR) are limited.

**Objectives:**

We aimed to investigate the association between the left atrial function index (LAFI) and outcome in patients undergoing TAVR.

**Methods:**

In this retrospective multicenter study, we assessed baseline LAFI in 733 patients undergoing TAVR for severe aortic stenosis in two German high-volume centers between 2008 and 2019. Based on receiver operating characteristic curves, patients were stratified according to their baseline LAFI into two groups (LAFI ≤ 13.5 vs. LAFI > 13.5) and assessed for post-procedural outcome. The primary endpoint of our study was the 1-year all-cause mortality.

**Results:**

Patients with a LAFI ≤ 13.5 had significantly more often atrial fibrillation (*p* < 0.001), lower LVEF (*p* < 0.001) and higher levels of NT-proBNP (*p* < 0.001). After TAVR, a significant improvement in the LAFI as compared to baseline was observed at 12 months after the procedure (28.4 vs. 32.9; *p* = 0.001). Compared to patients with a LAFI > 13.5, those with a LAFI ≤ 13.5 showed significantly higher rate of 1-year mortality (7.9% vs. 4.0%; *p* = 0.03). A lower LAFI has been identified as independent predictor of mortality in multivariate analysis (HR (95% CI) 2.0 (1.1–3.9); *p* = 0.03).

**Conclusion:**

A reduced LAFI is associated with adverse outcome and an independent predictor of mortality in TAVR patients. TAVR improves LAFI within 12 months after the procedure.

**Graphical abstract:**

Left Atrial Function Index (LAFI) in Patients undergoing Transcatheter Aortic Valve Implantation. **A** Kaplan–Meier survival analysis of 1-year all-cause mortality in patients with LAFI ≤ 13.5 compared with patients with LAFI > 13.5. Comparing rates of 1-year all-cause mortality between the different LAFI groups, we found a significant association between left atrial function and mortality. *LAFI* Left atrial function index. **B** Comparison of the mean LAFI before and after TAVR. After long-term follow-up the LAFI improved significantly. *LAFI* Left atrial function index; *FU* follow-up. **C** Assessment of the left atrial function index using the pre-procedural transthoracic echocardiography. **A** Measurement of the minimal left atrial volume (LAEDV). **B** Assessment of the maximal left atrial volume (LAESV).

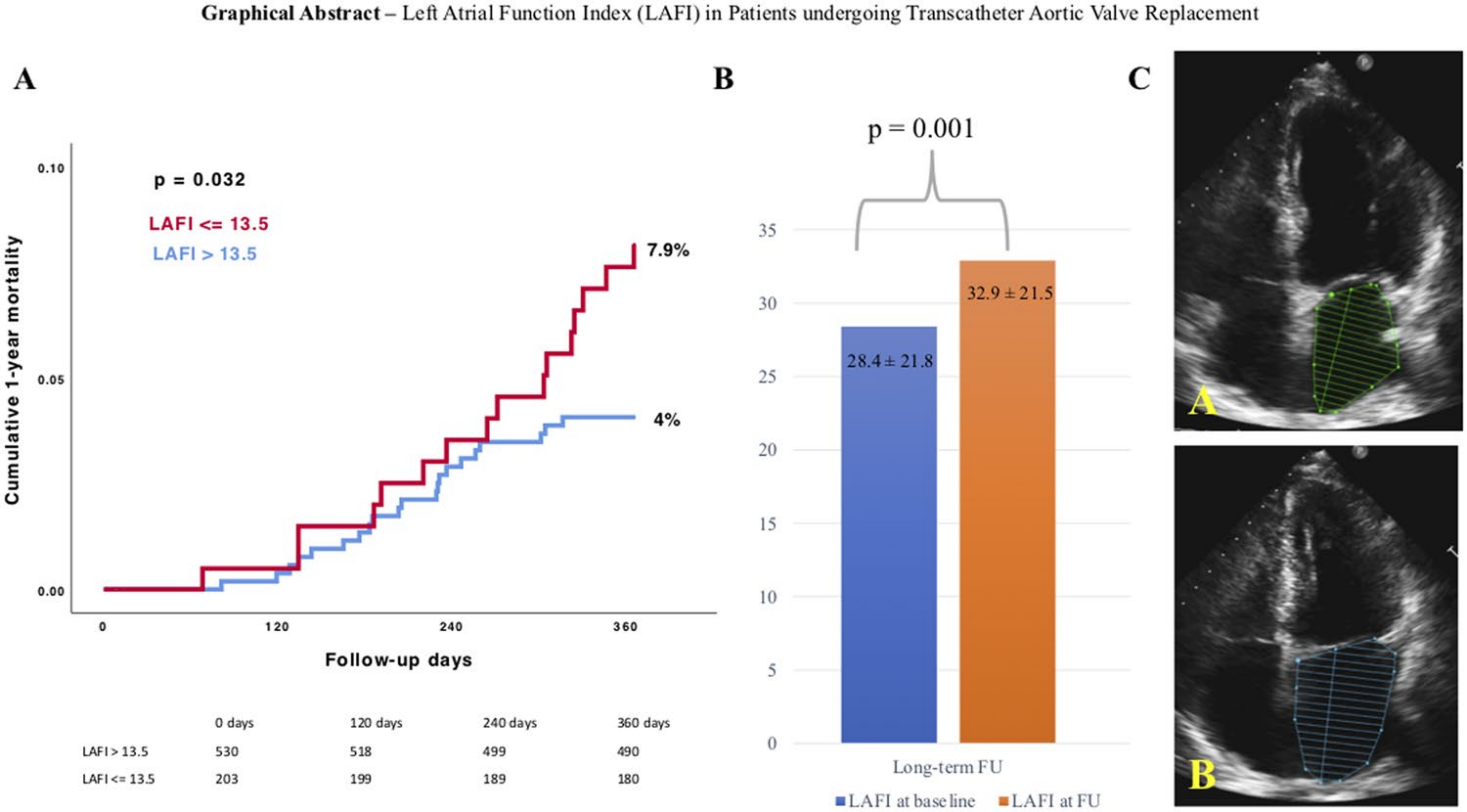

**Supplementary Information:**

The online version contains supplementary material available at 10.1007/s00392-022-02010-5.

## Introduction

Long-standing aortic stenosis is associated with pressure overload and subsequently concentric hypertrophy of the left ventricle, leading to increased atrial and ventricular filling pressures [1–3]. Consequently, severe aortic stenosis can result in myocardial remodeling and reduced left ventricular function, which has been shown to be associated with adverse outcome even after treatment [3–5]. As a consequence of increased filling pressures in aortic stenosis, atrial dilatation and impaired atrial function may occur [1–4]. The left atrial function index (LAFI) combines the reservoir function of the left atrium (LA), the adjusted LA volume and the stroke volume and is thus not only representing a marker of atrial function but also reflecting LV systolic and diastolic function [6–8]. The calculation of the LAFI is independent of the heart rhythm and has been shown to have a low inter—and intraobserver measurement variation [7].

In previous studies, the LAFI was a predictor of cardiovascular events in patient with heart failure [8] or a marker of improvement in patients with atrial fibrillation undergoing catheter ablation [9]. However, the applicability of the LAFI in patients with severe aortic stenosis has not been elucidated so far. Owing to its characteristics, the LAFI may represent a marker of the above described AS caused extraaortic cardiac damage associated with AS and might thus be a simple and useful echocardiographic parameter to optimize risk prediction.

In this retrospective multicenter study, we sought to investigate the association between the LAFI and outcome in patients undergoing TAVR and to evaluate its prognostic value to optimize risk prediction in this patient cohort.

## Methods

### Patient population

We retrospectively analyzed 733 patients with severe symptomatic aortic stenosis and increased surgical risk who underwent TAVR between February 2008 and April 2019 at the Heart Centers Bonn and Düsseldorf. The retrospective use of patient’s data for scientific needs was approved by the local ethics committee and written informed consent was obtained from all patients prior to the initial procedure.

Before the TAVR procedure, all patients underwent a careful evaluation including pre-interventional transthoracic and transesophageal echocardiography (including three-dimensional measurements) and had an interdisciplinary discussion with the local, institutional Heart Team. Details about patient screening, procedural techniques, and adjunctive medication have been described elsewhere [10–12].

**Fig. 1:**
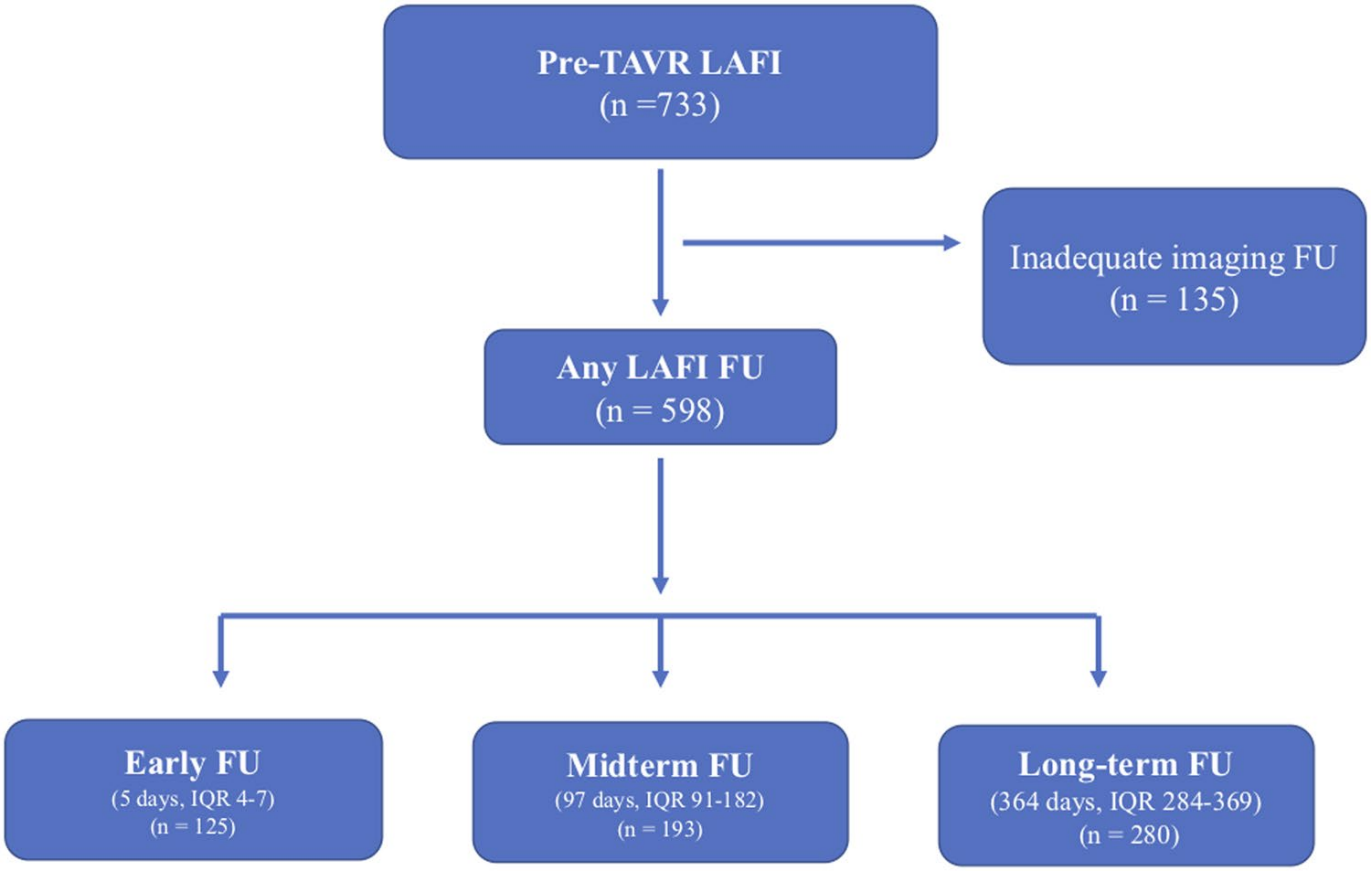
Study flow chart. *LAFI* Left atrial function index; *FU* follow-up

In this study, we only included patients with adequate baseline transthoracic echocardiography images, to allow a standardized assessment of the LAFI. As the LAFI has been established as rhythm-independent index [7], we also included patients with atrial fibrillation to our analysis. No other exclusion criteria were defined. To evaluate the effect of TAVR on LAFI we performed a subgroup analysis including 598 patients with adequate echocardiography images at different times of follow-up [pre-TAVR, post-TAVR before discharge (early follow-up), after 3-, or 6 months (midterm follow-up), or after 12 months of follow-up (long-term follow-up)], as shown in Fig. 1. Finally, we compared the LAFI before and after the procedure to investigate the impact of TAVR on the left atrial function. An improvement of the LAFI was defined as increase above the mean LAFI (i.e. > 28). In 342 patients with follow-up echocardiography images (6-, or 12 months), we had sufficient outcome data to evaluate 2-year all-cause mortality and in 588 patients we assessed 2-year all-cause mortality rates in accordance with the baseline LAFI.

### Left atrial function index (LAFI)

**Fig. 2:**
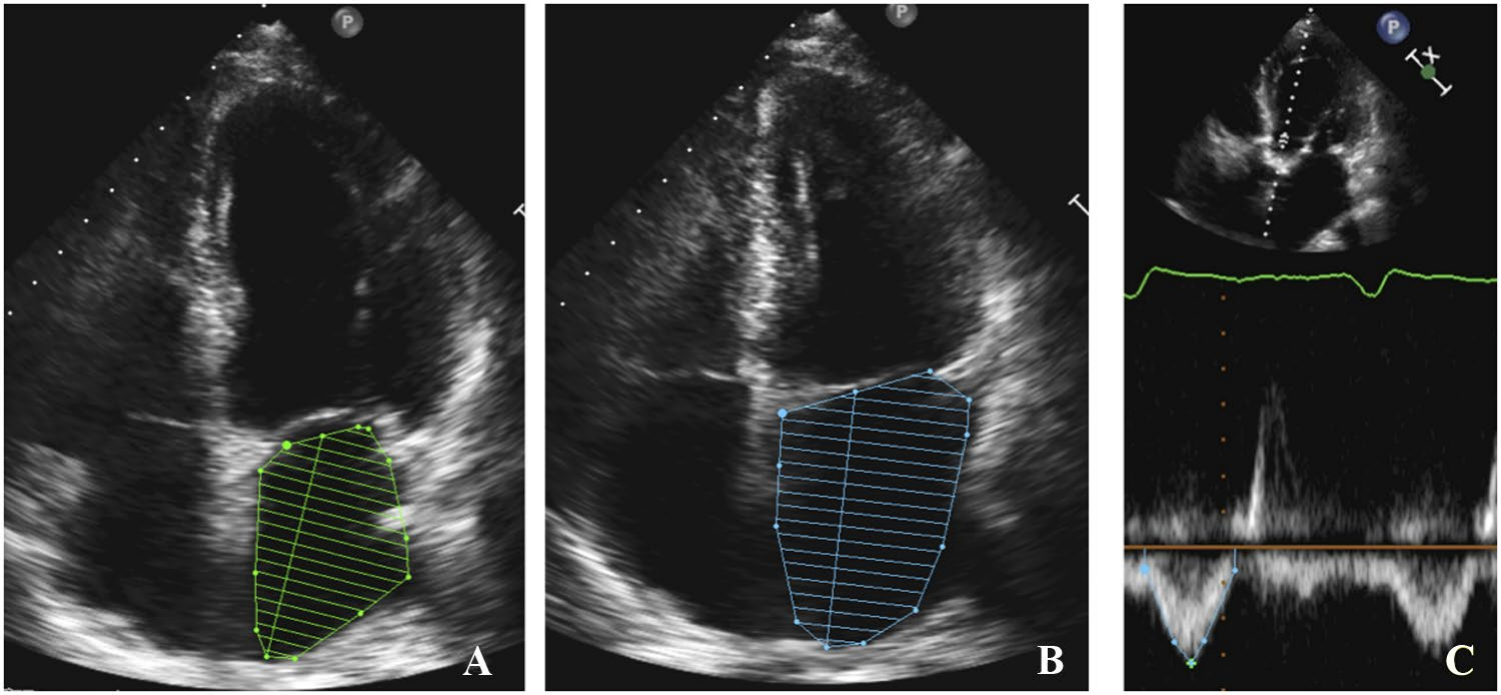
Echocardiographic assessment of the left atrial volume index. Assessment of the left atrial function index using the pre-procedural transthoracic echocardiography. **A** Measurement of the minimal left atrial volume (LAEDV). **B** Assessment of the maximal left atrial volume (LAESV). **C** Calculation of the velocity time integral across the left ventricular outflow tract (LVOT-VTI). *LAEDV* Minimal left atrial volume, *LAESV* maximal left atrial volume, *LVOT-VTI* outflow tract velocity time integral

To evaluate the LAFI, we retrospectively assessed several echocardiographic parameters using the pre- and postprocedural transthoracic echocardiography and determined the left atrial function index with the validated formula: LAFI = LAEF × LVOT–VTI/LAESVI (LAEF = LA emptying fraction, LAESVI = maximal LA volume indexed to the body surface area, LVOT-VTI = outflow tract velocity time integral). To calculate the LAEF we measured the left atrial maximal (LAESVi) and minimal (LAEDVi) indexed volumes and used the previously published formula: LAEF = ([LAESVi − LAEDVi]/LAESVi) × 100 [6–8] (Fig. 2). All echocardiographic parameters were assessed in accordance with the European Society of Cardiology (ESC) guidelines [13].

Since neither normal values nor established cut-offs for the LAFI in patients with severe aortic stenosis exist, we generated receiver operating characteristics (ROC) curves for 1-year mortality to determine the optimum cut-off value of the LAFI in our patient population. In consideration of the Youden-Index, a LAFI ≤ 13.5 was used to discriminate between the patients. Additionally, we categorized the LAFI according to quartiles, as previously reported [6, 7].

The retrospective analysis of echocardiographic images was performed by two independent cardiologists that were blinded to the outcome data.

### Study endpoints, data collection, and follow-up

The primary endpoint of our study was the 1-year all-cause mortality. In accordance to the VARC II criteria, we additionally assessed secondary endpoints: the occurrence of cardiovascular events like stroke or transient ischemic attack (TIA), myocardial infarction, pacemaker implantation and minor or major bleedings within 30 days after the TAVR procedure [14]. After patients were discharged, clinical follow-up data were prospectively collected during scheduled outpatient clinic visits or direct telephone interviews with the referring cardiologists, general practitioners, and patients.

### Statistical analysis

Data are presented as the mean ± standard deviation, if normally distributed, or as the median and an interquartile range (IQR) (quartile 1/quartile 3), if not normally distributed. Continuous variables were tested for having a normal distribution with the use of the Kolmogorov–Smirnov test. Categorical variables are given as frequencies and percentages. For continuous variables, a Student’s *t* test or a Mann–Whitney *U* test, as appropriate, was performed for comparing between two groups. When comparing more than two groups, ANOVA or the Kruskal–Wallis test was used. Spearman’s correlation coefficients were used to establish associations. The *χ*^2^ test was used for analysis of categorical variables. Survival according to the left atrial volume index was determined with use of the Kaplan–Meier method. The log-rank test was used to determine statistical differences in terms of survival. Finally, we performed a multivariate Cox proportional hazard regression analysis, which included univariate predictors with a *p*-value < 0.1, to assess independent predictors for 1-year all-cause mortality.

To compare the prognostic value of the LAFI for the prediction of the primary endpoint, receiver-operating characteristic (ROC) curves for 1-year mortality were generated to determine the optimum cut-off value. Based on the Youden Index, a LAFI of ≤ 13.5 was identified to provide best discrimination for outcome.

Statistical significance was assumed when the null hypothesis could be rejected at *p* < 0.05. Statistical analyses were conducted with PASW Statistics version 25.0.0.0 (IBM Corporation, Somers, NY, USA). The investigators initiated the study, had full access to the data, and wrote the manuscript. All authors vouch for the data and its analysis.

## Results

A total of 733 patients were included into the present analysis. The patient cohort presented with a mean age of 80.5 ± 7.1 years and an intermediate operative risk (EuroSCORE II: 4.6 (3.0/7.9); STS-PROM: 4.0 (2.7/6.2). Approximately half of the patients were male (48.7%) and all patients underwent transfemoral TAVR with the use of early and new generation transcatheter heart valves (THV) (early generation THV: 24.9% vs. new generation THV: 75.1%).

Mean LAFI was 28.0 ± 20.0 in the overall cohort, the median LAFI was 23.0 (IQR 13.0/39.0). Out of a total of 733 TAVR patients, 60.6% had a LAFI below the mean and 27.7% presented with a baseline LAFI ≤ 13.5, whereas 72.3% had a LAFI > 13.5. Follow-up outcome data were collected for all 733 patients, with a mean follow-up duration of 761 ± 674 days (median 430 days; IQR 365–1087).

### Baseline characteristics

Baseline characteristics according to the LAFI are presented in Table 1. Patients with a LAFI ≤ 13.5 had significantly more often atrial fibrillation (68.5% vs. 29.5%; *p* < 0.001), concomitant moderate to severe MR (54.2% vs. 35.9%; *p* < 0.001), moderate to severe TR (36.1% vs. 13.3%; *p* < 0.001) and presented at higher surgical risk. Additionally, patients with a LAFI ≤ 13.5 had a significantly lower left ventricular ejection fraction (LVEF) (50.0 ± 13.9% vs. 57.4 ± 11.6%; *p* < 0.001), a higher systolic pulmonary pressure (41.6 ± 17.2 mmHg vs. 31.2 ± 14.9 mmHg, *p* < 0.001) and higher levels of baseline NTpro-BNP (3372 pg/ml (IQR 1800–8766) vs. 1465 pg/ml (IQR 592–3475); *p* < 0.001).

**Table 1:** Baseline characteristics according to the baseline LAFI

	All patients *n* = 733	LAFI ≤ 13.5 *n* = 203	LAFI > 13.5 *n* = 530	*p*-value
Age, ± SD	80.3 ± 6.9	80.3 ± 8.5	80.3 ± 6.2	0.99
BMI, ± SD	27.3 ± 6.2	27.0 ± 8.1	27.4 ± 5.3	0.40
Male sex, %	357 (48.7)	107 (52.7)	250 (47.2)	0.18
Peripheral artery disease, *n*	238 (38.4)	71 (43)	167 (36.7)	0.15
COPD, *n*	157 (21.4)	43 (21.2)	114 (21.5)	0.921
Hypertension, *n*	647 (88.4)	177 (87.2)	470 (88.8)	0.53
Diabetes, *n*	231 (31.6)	65 (32.0)	166 (31.4)	0.86
NYHA IV, *n*	58 (7.9)	20 (9.9)	38 (7.2)	0.23
Previous MI, *n*	130 (17.7)	42 (20.7)	88 (16.6)	0.19
Atrial fibrillation, *n*	295 (40.2)	139 (68.5)	156 (29.5)	< 0.001
MR ≥ moderate, *n*	300 (41.0)	110 (54.2)	190 (35.9)	< 0.001
TR ≥ moderate, *n*	121 (19.4)	60 (36.1)	61 (13.3)	< 0.001
Ejection fraction, %	55.2 ± 12.8	50.0 ± 13.9	57.4 ± 11.6	< 0.001
sPAP, mmHg	34.5 ± 16.4	41.6 ± 17.2	31.2 ± 14.9	< 0.001
NT-proBNP, pg/ml	1949 (785/4683)	3372 (1800/8766)	1465 (592/3475)	< 0.001
EuroSCORE	16.7 (10.1/26.2)	20.6 (12.3/33.4)	15.2 (9.7/23.3)	< 0.001
EuroSCORE II	4.6 (3.0/7.9)	6.0 (3.5/10.1)	4.1 (2.5/6.9)	< 0.001
STS-Score	4.0 (2.7/6.2)	4.6 (3.1/7.0)	4.0 (2.5/6.0)	0.001
AV *P*_mean_, mmHg	40 ± 14.7	38.7 ± 13.3	39.3 ± 17.5	0.32
AV *V*_max_, m/s	3.9 ± 0.8	3.8 ± 0.9	4.0 ± 0.7	0.32
AVA, cm^2^	0.7 ± 0.18	0.7 ± 0.18	0.7 ± 0.2	0.33
Aortic regurgitation post-TAVR				0.09
Grade 0	374 (52.1)	104 (51.7)	270 (52.2)	
Grade 1	321 (44.7)	86 (42.8)	235 (45.5)	
Grade 2	23 (3.2)	11 (5.5)	12 (2.3)	
Contrast media, ml	135.95 ± 51.3	139.1 ± 54.9	134.7 ± 49.7	0.22
Fluoroscopy time, min	20.6 ± 9.5	20.4 ± 9.1	20.6 ± 9.7	0.53
Procedure time, min	72.3 ± 30.9	70.9 ± 35.4	72.8 ± 29.1	0.64

Values are mean (± SD), median (IQR 1/3) or *n*/*N* (%)

*BMI* body mass index, *COPD* chronic obstructive pulmonary disease, *NYHA* New York Heart Association, *MI* myocardial infarction, *MR* mitral regurgitation, *TR* tricuspid regurgitation, *sPAP* systolic pulmonary artery pressure, *NT-proBNP* n-terminal pro brain natriuretic peptide, *EuroSCORE* European System for Cardiac Operative Risk Evaluation, *STS-Score* the Society Thoracic of Surgeons-Score, *AV* *P*_*max*_ aortic valve maximum pressure, *AV* *P*_*mean*_ aortic valve mean pressure, *AV* *V*_*max*_ peak aortic valve jet velocity, *AVA* aortic valve area

The aortic valve area (AVA), the peak aortic jet velocity (*V*_max_) and the mean pressure gradient (*P*_mean_) did not differ significantly before TAVR procedure between the LAFI groups.

Procedural characteristics such as the amount of contrast media, the fluoroscopy or procedure time did not differ significantly between the groups. The procedure was successful in all study participants and the postinterventional rate of paravalvular leakage was comparable between the groups (*p* = 0.09).

### Clinical outcomes

**Fig. 3:**
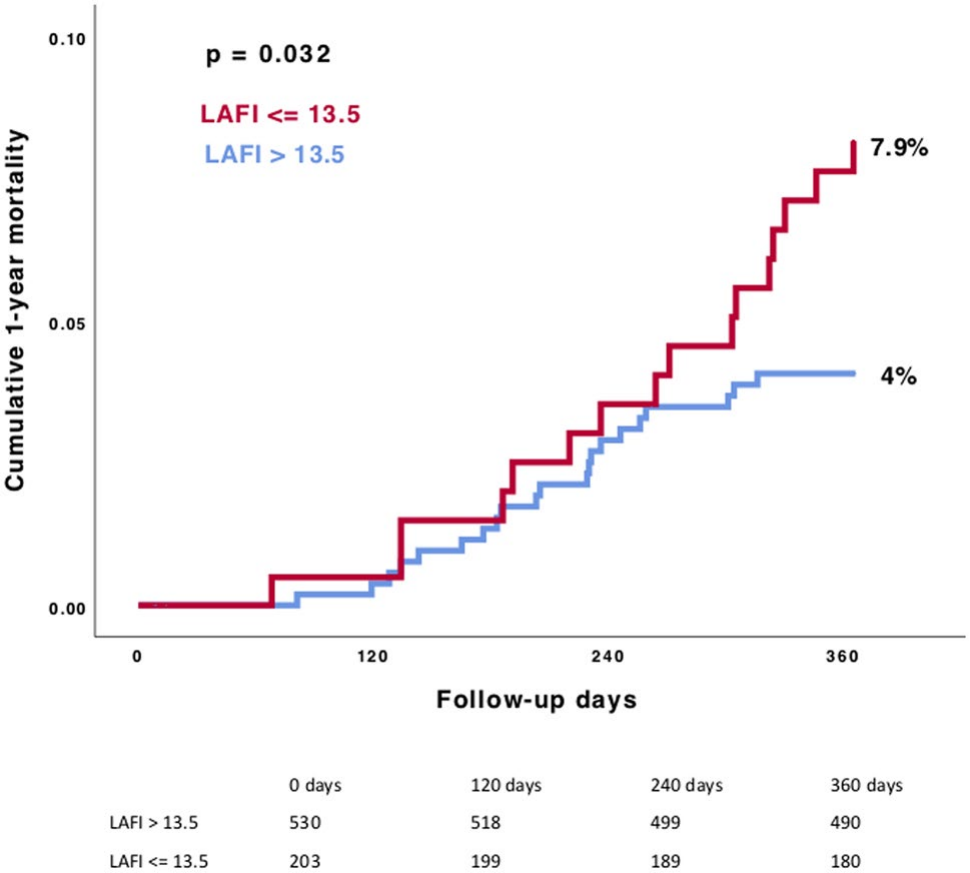
Kaplan–Meier survival analysis of 1-year all-cause mortality in patients with LAFI ≤ 13.5 compared with patients with LAFI > 13.5. Comparing rates of 1-year all-cause mortality between the different LAFI groups, we found a significant association between left atrial function and mortality. *LAFI* Left atrial function index

Outcome data according to the baseline LAFI are presented in Table 2. We found higher 1-year mortality rates in patients with a lower LAFI (7.9% vs. 4.0%; *p* = 0.03), as presented in Fig. 3. Categorizing patients according to LAFI quartiles did not further improve discrimination of mortality (Supplementary Appendix, Fig. 1)*.* In 588 patients we had sufficient outcome data to evaluate the 2-year all-cause mortality rate. The Kaplan–Meier survival analysis showed even after 2 years of follow-up a significant association between a reduced LAFI and higher mortality rates (16.5% vs. 9.8%; *p* = 0.02) (Supplementary Appendix, Fig. 2).

**Table 2:** Outcome according to the baseline LAFI

	All patients *n* = 733	LAFI ≤ 13.5 *n* = 203	LAFI > 13.5 *n* = 530	*p*-value
1-year all-cause mortality, %	37 (5.1)	16 (7.9)	21 (4.0)	**0.032**
Stroke or TIA within 30 days, %	11 (1.5)	2 (1.0)	9 (1.7)	0.47
Pacemaker implantation within 30 days, %	105 (14.3)	28 (13.8)	77 (14.5)	0.91
Myocardial infarction within 30 days, %	–	–	–	–
Major bleeding within 30 days, %	23 (3.1)	7 (3.4)	16 (3.0)	0.76
Minor bleeding within 30 days, %	132 (18.1)	33 (16.3)	99 (18.8)	0.43

Values are *n*/*N* (%)

Statistically significant difference is given in bold

*LAFI* left atrial function index, *TIA* transient ischemic attack

The secondary endpoints did not differ significantly between the groups (Table 2).

In multivariate analysis, the LAFI has been identified as the only independent predictor of mortality (HR (95% CI) 2.0 (1.1–3.9); *p* = 0.03), as shown in Table 3. Interestingly, concomitant atrial fibrillation was not significantly associated with mortality in univariate analysis.

**Table 3:** Multivariate analysis with the most important confounders

	Univariate analysis HR (95% CI)	*p* value	Multivariate analysis HR (95% CI)	*p* value
Chronic kidney injury	1.3 (0.6–2.5)	0.49	–	–
COPD	0.8 (0.4–1.9)	0.69	–	–
Ejection fraction	1.0 (0.9–1.1)	0.84	–	
Diabetes	1.5 (0.7–2.8)	0.25	–	–
PAD	1.3 (0.6–2.7)	0.39	–	–
Atrial fibrillation	1.6 (0.8–2.9)	0.17	–	–
MR ≥ moderate	1.7 (0.9–3.2)	0.10	–	–
TR ≥ moderate	1.8 (0.9–4.0)	0.10	–	–
sPAP	1.0 (0.9–1.1)	0.21	–	–
NT-proBNP	1.0 (1.0–1.0)	0.28	–	–
Logistic EuroSCORE	1.0 (0.9–1.1)	0.17	–	–
STS Prom	1.0 (0.8–1.2)	**0.06**	1.0 (0.9–1.1)	0.12
Aortic regurgitation post-TAVR	0.7 (0.3–1.3)	0.27	–	–
NYHA IV	1.8 (0.7–4.7)	0.20	–	–
LAFI at baseline	2.0 (1.0–3.8)	**0.03**	2.0 (1.1–3.9)	**0.03**

Statistically significant differences are given in bold

*HR* hazard ratio, *CI* confidence interval, *COPD* chronic obstructive pulmonary disease, *PAD* peripheral artery disease, *TAVR* transcatheter aortic valve replacement, *NYHA* New York Heart Association, *LAFI* left atrial function index, *NT-proBNP* n-terminal pro brain natriuretic peptide, *EuroSCORE* European System for Cardiac Operative Risk Evaluation, *STS-Score* the Society Thoracic of Surgeons-Score, *MR* mitral regurgitation, *TR* tricuspid regurgitation, *sPAP* systolic pulmonary artery pressure

### Impact of TAVR on LAFI

**Fig. 4:**
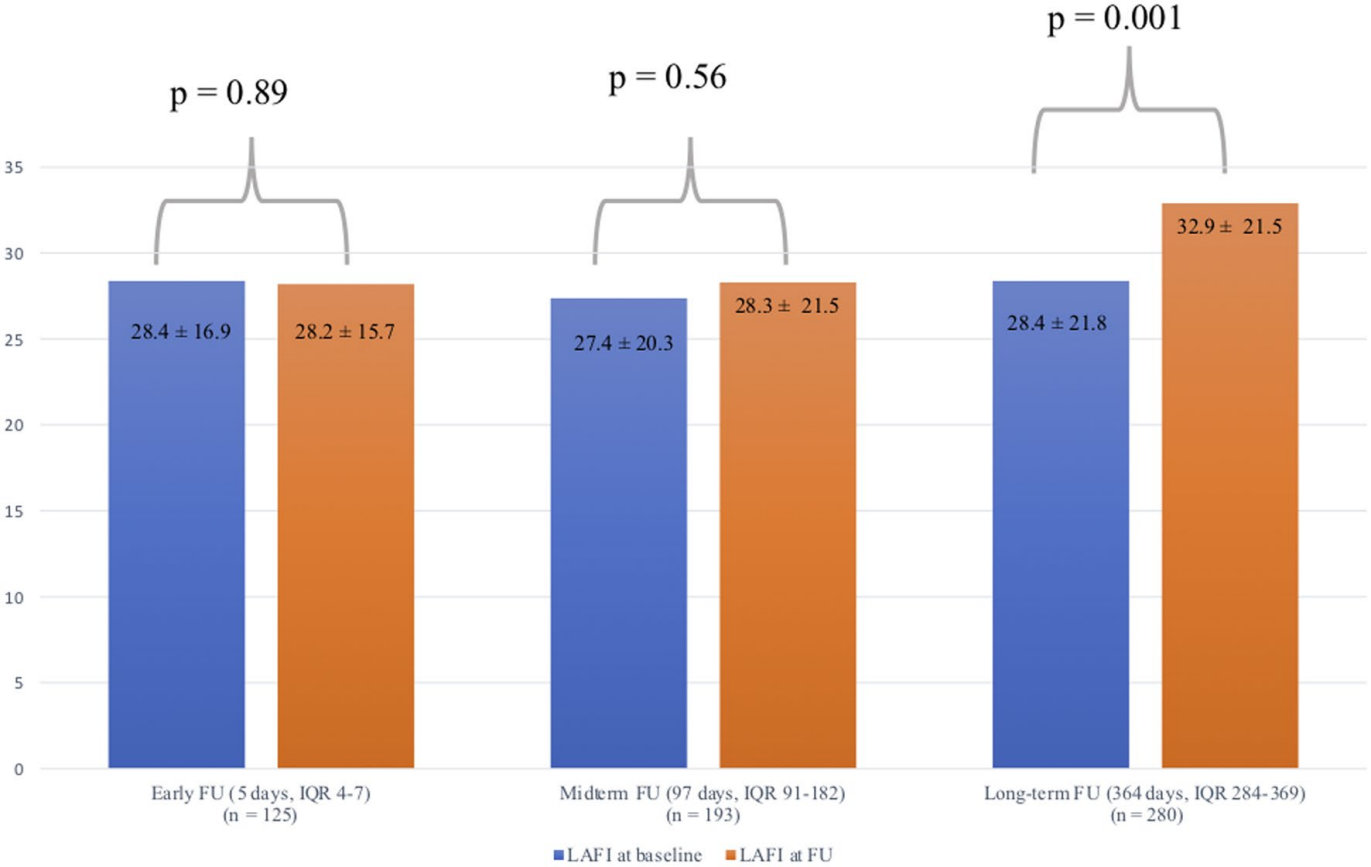
Comparison of the mean LAFI before and after TAVR. After long-term follow-up the LAFI improved significantly. *LAFI* Left atrial function index, *FU* follow-up

In 598/733 patients (81.5%), imaging follow-up was available to calculate the LAFI after the TAVR procedure (Fig. 1). A comparison of the mean LAFI before and after TAVR showed a significant improvement of the left atrial function after 12 months of follow-up (28.4 ± 21.8 vs. 32.9 ± 21.5; *p* = 0.001), while no significant differences were observed at earlier time points (i.e. 3 or 6 months post-TAVR) (Supplementary Appendix, Table 1). At the early or midterm follow-up, the improvement of the LAFI was not significant (Fig. 4). Interestingly, patients who showed an improvement of LAFI in follow-up echocardiography, presented with significantly lower left ventricular function (*p* = 0.001) and higher rates of atrial fibrillation (*p* = 0.004) at baseline compared to patients without LAFI improvement (Table 4).

**Table 4:** Baseline characteristics according to LAFI improvement at any time of FU

	Patients with LAFI improvement at any FU *n* = 89	Patients without LAFI improvement at any FU *n* = 508	*p*-value
Age, ± SD	79.3 ± 6.9	80.4 ± 7.9	0.25
Male sex, %	46 (51.7)	242 (47.6)	0.48
AV *P*_mean_, mmHg	36.9 (11.2)	40.0 (16.5)	0.59
AV *V*_max_, m/s	4.0 ± 0.6	4.0 ± 0.8	0.92
AVA, cm^2^	0.69 ± 0.2	0.73 ± 0.1	0.07
Ejection fraction, %	50.8 ± 14.0	56.1 ± 12.6	**0.001**
Atrial fibrillation, *n*	48 (53.9)	191 (37.6)	**0.004**
Aortic regurgitation post-TAVR, *n*			0.06
Grade 0	45 (51.7)	275 (54.7)	
Grade 1	36 (41.4)	217 (43.1)	
Grade 2	6 (6.9)	11 (2.2)	

Values are mean (± SD), median (IQR 1/3) or *n*/*N* (%)

Statistically significant differences are given in bold

*FU* follow-up, *LAFI* left atrial function index, *AV* *P*_*mean*_ aortic valve mean pressure, *AV* *V*_*max*_ peak aortic valve jet velocity, *AVA* aortic valve area, *TAVR* transcatheter aortic valve replacement

**Fig. 5:**
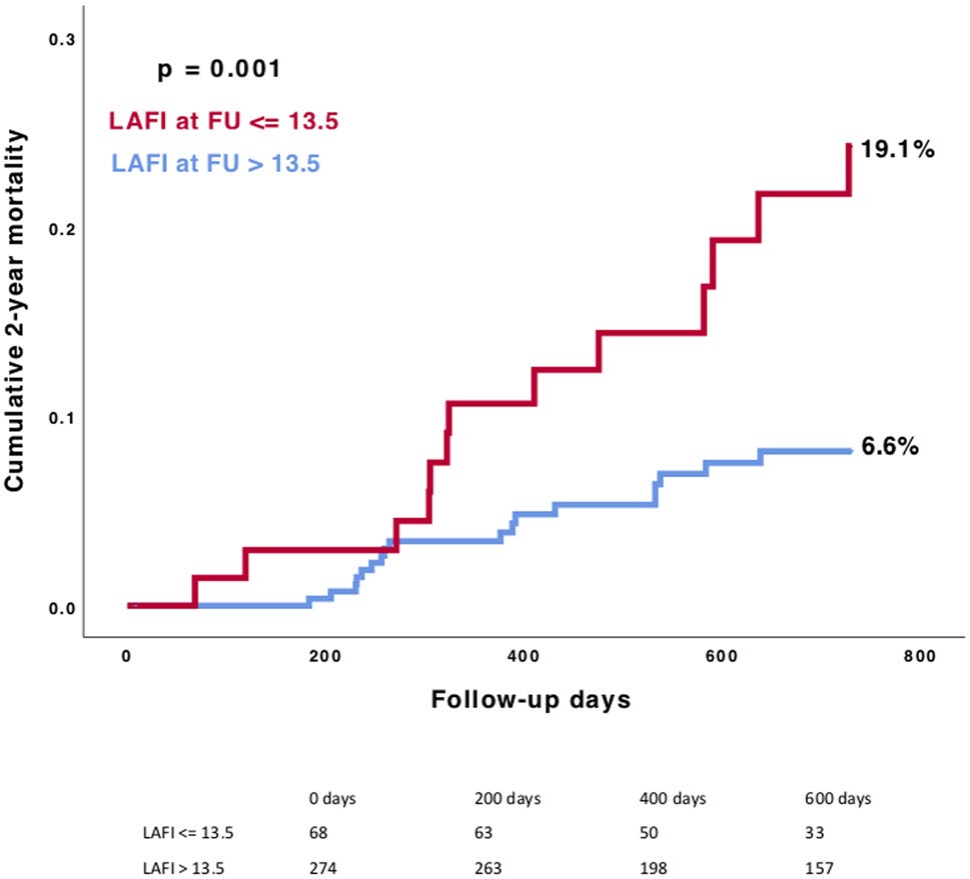
Kaplan–Meier survival analysis of 2-year all-cause mortality according to the two LAFI groups including patients with midterm follow-up echocardiography images. The LAFI at follow-up showed significant association with 2-year all-cause mortality in the Kaplan–Meier survival analysis. *LAFI* Left atrial function index

In 342/733 patients (46.6%) we had follow-up echocardiography images at 6-, or 12-months and evaluated 2-year mortality rates. The Kaplan–Meier survival analysis revealed significantly higher rates of mortality in patients with a lower LAFI ≤ 13.5 (19.1% vs. 6.6%; *p* = 0.001) (Fig. 5).

## Discussion

To the best of our knowledge, this is the first study evaluating the applicability of the left atrial function index and its association with outcome in patients with severe aortic stenosis undergoing transcatheter aortic valve replacement. The main results of our study are as follows:

1. A lower LAFI was associated with adverse outcome in patients undergoing TAVR; a LAFI cut-off of ≤ 13.5 was identified to be associated with mortality and has been shown to be an independent predictor of mortality.
2. LAFI improved in long-term follow-up echocardiography after TAVR.
3. A low LAFI in mid-term follow-up echocardiography after TAVR procedure was significantly associated with mortality.

### Left atrial function index

Longstanding AS causes pressure overload of the left ventricle resulting in left ventricular (LV) hypertrophy and increasing filling pressures of both, the LV and LA. As a consequence, myocardial remodeling can occur und result in impaired LV and LA function [1–4, 15–19]. In this connection, the term ‘atrial cardiomyopathy’ could be applied. A concept that has been introduced 2016 by Goette et al*.* to describe “structural, architectural, contractile or electrophysical changes affecting the atria with the potential to produce clinically-relevant manifestations” [20]. Many structural heart diseases have been shown to cause atrial cardiomyopathy due to hemodynamic changes, including heart valve diseases.

The assessment of LV and LA dysfunction in the context of cardiovascular diseases was the objective of several previous studies [3–9], and one of the possible quantification methods to evaluate not only LA but especially the LV function has been shown to be the left atrial function index [7–9]. The LAFI considers the reservoir function of the LA, the indexed LA volume as well as the cardiac output and thus permits conclusions on the LV systolic and diastolic function [8]. The calculation of the LAFI is based on rhythm-independent parameters, which means that it can be used in patients with atrial fibrillation or atrial flutter. An important benefit considering the high prevalence of these cardiac arrhythmias. Another advantage of the LAFI is the simple acquisition using only three parameters of a standard transthoracic echocardiography.

In our study, we evaluated the applicability of the LAFI in patients with symptomatic severe AS undergoing TAVR. To this point, no specific cut-off or normal value has been described regarding the LAFI in patients with AS. However, in patients with chronic heart failure, the median LAFI has been described to be 16.56 [8] while in patients with atrial fibrillation, a LAFI > 30 was considered to represent a normal atrial function [9]. In contrast, in a study by Wong et al. the mean LAFI in patients with coronary heart disease was 40.1 [21]. In our cohort, the mean LAFI was 23, while the median was 28 and almost 2/3 of the TAVR patients in our study presented with a baseline LAFI below the mean. This result is not surprising considering the hemodynamic effects of long-standing aortic stenosis on remodeling of both the left ventricle and atrium [15–19], leaving the LAFI in aortic stenosis somewhat in between that of patients with chronic heart failure and those with coronary artery disease.

### LAFI and outcome

In previous studies, the LAFI has already been shown to be negatively associated with adverse outcome in patients with cardiovascular disease. Sargento et al*.* demonstrated that the LAFI is a predictor of long-term survival in clinically stable and optimally treated outpatients with heart failure (HF) with reduced left ventricular ejection fraction (HFrEF). Even after adjustment for the heart rhythm, the LAFI remained a significant predictor of survival in this patient population [8]. Wong et al*.* investigated the LAFI and the risk of incident ischemic stroke or transient ischemic attack (TIA). The authors found a significant association between the LAFI and the risk of incident ischemic stroke or TIA [21]. In another analysis of patients with preserved baseline ejection fraction, the LAFI independently predicted HF hospitalization in these subjects [22]. However, the applicability of the LAFI and its association with outcome in patients with severe AS undergoing TAVR has not been elucidated. Against this background, we assessed the LAFI in 733 TAVR patients and could demonstrate that a lower LAFI (≤ 13.5) was significantly associated with higher rates of 1-year all-cause mortality and that the LAFI was even an independent predictor of mortality. Since echocardiography is still deemed to be the gold standard for the diagnosis and therapeutic decision-making in patients with severe aortic stenosis, the assessment of the LAFI in this patient cohort is not associated with extra efforts or costs. On the contrary, our study results indicate that the assessment of LAFI allows for risk prediction in this patient cohort and provides additional information in term of risk stratification.

Our study population represented a real-world patient cohort, with advanced age and a median logistic EuroSCORE of 16.7 and STS score of 4.0, representing an intermediate operative risk for the patient. The overall 1-year mortality rate in our study cohort was 5.0%. This result corresponds to a very low all-cause mortality rate compared to the large Placement of Aortic Transcatheter Valves (PARTNER) 2 cohort A randomized trial, comparing TAVR versus surgical aortic valve repair in intermediate risk patients, which reported an overall 1-year mortality rate of 12.3 in the TAVR cohort [23]. This finding might represent a potential bias in our cohort and future studies are necessary to validate our results.

### Recovery of the LAFI after TAVR

In our study population, the LAFI improved in long-term echocardiography after TAVR. These data are consistent with previous studies. Following TAVR a significant improvement in atrial reservoir and conduit function could be observed [18] and also an increase of LA reservoir function and decrease of LA volume after surgical aortic valve repair (SAVR) has been described in the past [22]. A hypothetical approach to explain the recovery of the LAFI after aortic valve repair, might be the reduced LA dilation and thus the lower expression of ADAMs (A Disintegrin and Metalloproteinase). Previous studies have demonstrated that the expression of ADAMs is associated with LA dilatation in patients with atrial fibrillation [24]. A similar pathogenic mechanism might underlie in patients with AS and at least partly explain the recovery of the LAFI due to a reversible atrial dilatation.

But also, the single parameters included in the LAFI have been found to improve after TAVR (e.g. LVEF and stroke volume) [25]. In our study, an improvement of the LAFI was found after long-term FU (i.e. approximately 12 months). However, patients with a lower LAFI in follow-up echocardiography showed still a significant worse outcome, compared to patients with a higher LAFI (> 13.5). Our data suggest that the hemodynamic effects of long-standing aortic stenosis on myocardial remodeling are at least partly reversible.

### Study limitations

Limitations of our study are sample size and its retrospective character. Furthermore, a certain selection and therapy bias might have impacted our results, since only patients who underwent a TAVR procedure have been included. Another limitation is the fact that we had no functional outcome parameter like the NYHA class or NT-proBNP value at follow-up available to evaluate the functional outcome in accordance to the LAFI. Therefore, the results of this study should be considered hypothesis-generating. Prospective and larger trials are necessary to further evaluate the clinical importance of the LAFI in patients with severe aortic stenosis undergoing a TAVR procedure.

## Conclusion

The left atrial function index represents a promising marker of LV and LA dysfunction in patients undergoing transcatheter aortic valve replacement. An impaired LAFI is significantly associated with adverse outcome and an independent predictor of 1-year all-cause mortality in this patient cohort. TAVR has a positive impact on LAFI in long-term follow-up.

## Supplementary Information

Below is the link to the electronic supplementary material.

Supplementary file1 (DOCX 185 kb)

## Abbreviations

TAVR: Transcatheter aortic valve replacement
LAFI: Left atrial function index
AS: Aortic valve stenosis
LA: Left atrium
LAEF: LA emptying fraction
LVOT-VTI: Outflow tract velocity time integral
LAESVi: Maximal LA volume indexed to the body surface area
LAEDVi: Minimal LA volume indexed to the body surface area
IQR: Interquartile range
COPD: Chronic obstructive pulmonary disease
LVEF: Left ventricular ejection fraction
SAVR: Surgical aortic valve replacement
STE: Speckle tracking echocardiography
