# Supplementary material for: Left atrial function index (LAFI) and outcome in patients undergoing transcatheter aortic valve replacement
Source: Clin Res Cardiol. 2022 Mar 23;111(8):944–54. doi: 10.1007/s00392-022-02010-5 (PMC9334426; doi:10.1007/s00392-022-02010-5)
Supplement: Supplementary file 1 — Supplementary file1 (DOCX 185 kb) [file 392_2022_2010_MOESM1_ESM.docx]

**Supplementary Appendix**

**Figure 1** – Kaplan-Meier survival analysis of 1-year all-cause mortality according to LAFI quartiles

Kaplan-Meier survival analysis of 1-year all-cause mortality according to LAFI quartiles.

Comparing rates of one-year all-cause mortality between the different LAFI quartiles, we could not find a significant association between left atrial function and mortality.

*Left atrial function index = LAFI*

**Figure 2** *–* Kaplan-Meier survival analysis of 2-year all-cause mortality in patients with LAFI ≤ 13.5 compared with patients with LAFI > 13.5.

Comparing rates of two-year all-cause mortality between the different LAFI groups in patients with available 2-year FU data, higher mortality was observed in patients with lower LAFI.

*Left atrial function index = LAFI*

**Table 1 –** Comparison of mean LAFI before and after TAVR

|  | Mean LAFI at baseline | Mean LAFI at FU | p-value |
| --- | --- | --- | --- |
| FU at 12 months (n = 280) | 28.4 ± 21.8 | 32.9 ± 21.5 | **0.001** |
| FU at 6 months (n = 62) | 29.9 ± 19.9 | 32.7 ± 21.4 | 0.28 |
| FU at 3 months (n = 131) | 26.2 ± 20.5 | 26.2 ± 21.3 | 0.99 |
| FU at discharge (n = 125) | 28.4 ± 16.9 | 28.2 ± 15.7 | 0.89 |

*Values are mean (± SD)*

*LAFI = left atrial function index; FU = follow-up*
